# Integration of next-generation sequencing in clinical diagnostic molecular pathology laboratories for analysis of solid tumours; an expert opinion on behalf of IQN Path ASBL

**DOI:** 10.1007/s00428-016-2025-7

**Published:** 2016-09-27

**Authors:** Zandra C Deans, Jose Luis Costa, Ian Cree, Els Dequeker, Anders Edsjö, Shirley Henderson, Michael Hummel, Marjolijn JL Ligtenberg, Marco Loddo, Jose Carlos Machado, Antonio Marchetti, Katherine Marquis, Joanne Mason, Nicola Normanno, Etienne Rouleau, Ed Schuuring, Keeda-Marie Snelson, Erik Thunnissen, Bastiaan Tops, Gareth Williams, Han van Krieken, Jacqueline A Hall, On behalf of IQN Path ASBL

**Affiliations:** 1 UK NEQAS for Molecular Genetics, Department of Laboratory Medicine, Royal Infirmary of Edinburgh, Edinburgh, EH16 4SA UK; 2 i3S Instituto de Investigação e Inovação em Saúde/IPATIMUP Institute of Molecular Pathology and Immunology, University of Porto, Porto, Portugal; 3 Department of Pathology, University Hospital Coventry and Warwickshire, Coventry, CV2 2DX UK; 4 Biomedical Quality Assurance Research Unit, Department of Public Health and Primary Care, KU Leuven-University of Leuven, Leuven, Belgium; 5 Clinical Pathology, Laboratory Medicine, Medical Services, Region Skåne, Lund, Sweden; 6 Genomics England, Queen Mary University of London, Dawson Hall, Charterhouse Square, London, EC1M 6BQ UK; 7 Institute of Pathology, Berlin, Germany and the DGP, German Society of Pathology, Charite, University Medicine Berlin, Berlin, Germany; 8 Department of Pathology and Department of Human Genetics, Radboud University Medical Center, Nijmegen, The Netherlands; 9 Oncologica UK Ltd, Suite 15-16, The Science Village, Chesterford Research Park, Cambridge, CB10 1XL UK; 10 Center of Predictive Molecular Medicine, CeSI-MeT, University of Chieti, Chieti, Italy; 11 Cell Biology and Biotherapy Unit, Istituto Nazionale Tumouri “Fondazione Giovanni Pascale” IRCCS, Naples, Italy; 12 Department of Medical Biology and Pathology, Genetic and Pathology Molecular Service, Gustave Roussy, 114 Rue Edouard Vaillant, 94800 Villejuif, France; 13 Department of Pathology, University of Groningen, University Medical Center of Groningen, Groningen, The Netherlands; 14 Pathology, VU University Medical Center, Amsterdam, the Netherlands; 15 International Quality Network for Pathology (IQN Path) Association Sans But Lucratif (A.S.B.L), 17 Boulevard Royal, L2449 Luxembourg City, Luxembourg; 16 Division of Cancer, Department of Surgery and Cancer, Imperial College London, London, UK

**Keywords:** Next-generation sequencing, Best practice, Molecular pathology, Solid tumours, Quality

## Abstract

The clinical demand for mutation detection within multiple genes from a single tumour sample requires molecular diagnostic laboratories to develop rapid, high-throughput, highly sensitive, accurate and parallel testing within tight budget constraints. To meet this demand, many laboratories employ next-generation sequencing (NGS) based on small amplicons. Building on existing publications and general guidance for the clinical use of NGS and learnings from germline testing, the following guidelines establish consensus standards for somatic diagnostic testing, specifically for identifying and reporting mutations in solid tumours. These guidelines cover the testing strategy, implementation of testing within clinical service, sample requirements, data analysis and reporting of results. In conjunction with appropriate staff training and international standards for laboratory testing, these consensus standards for the use of NGS in molecular pathology of solid tumours will assist laboratories in implementing NGS in clinical services.

**Electronic supplementary material:**

The online version of this article (doi:10.1007/s00428-016-2025-7) contains supplementary material, which is available to authorized users.

## Introduction

The clinical demand for mutation detection within multiple genes from a single tumour sample requires molecular diagnostic laboratories to develop rapid, high-throughput, highly sensitive, accurate and parallel testing within tight budget constraints. To meet this demand, many laboratories employ next-generation sequencing (NGS) based on small amplicons. Solid tumour sequencing using NGS brings specific challenges; the samples available are often small, particularly if coming from diagnostic biopsies, and so the DNA quantity available for testing may be small. The samples may not have been optimally treated for NGS, formalin-fixed, paraffin-embedded (FFPE) samples are particularly challenging and there can be considerable variation in the processing of material throughout the fixation process. The neoplastic cell, stromal and necrotic content can be highly variable within a sample, and even expert pathologists can judge the purity and suitability for testing very differently. Tumour heterogeneity can cause particular challenges as low-level mutations may not be present in all cells and high-sensitivity techniques are required to reliable call low-level variants.

The clinical utility of NGS as a tool for use in clinical practice is now becoming apparent. As NGS opens the possibility for more extensive tumour profiling, this increases the opportunities for patients to access different drugs. For example, a study in colorectal cancer found that use of NGS for genotyping beyond *KRAS* enabled selection of the best treatment currently available for more than half of the patients profiled [1]. NGS has also enabled new clinical trial designs such as umbrella trials, which require patient stratification through enabling efficient genomic profiling for patient enrollment. Examples of studies include FOCUS4 and WINTHER, as well as basket trials such as MATCH [2]. The results from the SHIVA study were entirely based on the use of an NGS panel of actionable genes [3]. Several panels have been validated for clinical use such as a 22-gene panel for lung and colorectal [4] and a targeted NGS assay for detecting somatic variants in non-small cell lung, melanoma and gastrointestinal malignancies [5]. Experience suggests that as methods improve, the use of FFPE material will likely not be a limitation for routine testing [6]. Finally, the cost-effectiveness of a targeted NGS approach has been recently stressed in the fourth-line treatment of metastatic lung carcinoma and should be extendable to other cancer [7]. All these results indicate the clinical utility and application of NGS profiling of tumours in the clinic.

The following guidelines aim to establish consensus standards for somatic diagnostic testing rather than germline testing, specifically for identifying and reporting mutations in solid tumours. Consensus standards will include the testing strategy, implementation of testing within clinical service, sample requirements, data analysis and reporting of results. In addition, laboratories providing NGS clinical diagnostic testing must adhere to recognised International Standards [8, 9] and staff must be appropriately qualified, trained and competent.

## Testing strategies

Strategies for molecular pathology testing are dictated according to the purpose of the test. However, current ESMO and AMP clinical practice guidelines approve only a limited number of predictive and prognostic biomarkers for clinical use, listed in [10, 11]. At the same time, there has been a steady increase in clinical trials that select patients based on their molecular tumour profiles suggesting that new therapeutics will soon require patient selection on this basis. Hence, concomitant analysis of multiple genes in different tumour types is increasingly important for both differential diagnostics and prediction of response to targeted therapies. This will drive the development of comprehensive diagnostic panels that detect multiple gene mutations which may be used for multiple tumour types. Such tests could rely on primer-based amplification or probe-based capture methods, followed by NGS and bioinformatic analyses to define genomic alterations.

The number and scope of genes to be tested depend on the purpose of the testing. For example, for companion diagnostic use, the number of genes currently recommended for clinical testing is very limited [10, 11] and will also depend on the availability of targeted treatments and reimbursement schemes and will vary from country to country. However, if there are clinical trials open in that country for which NGS panel test results can be used to stratify patients into studies, then a broader range of genes might be tested.

Currently, the test method of choice is an assay that detects a panel of clinically actionable genomic alterations at specific gene-coding regions, so defined by the clinical diagnosis and/or availability of targeted drug therapies. There is an increasing interest in extending these panels to include genomic alterations associated with acquired resistance to target-based agents, which will become increasingly important as new drugs become available e.g. acquired mutations of *EGFR* such as p.(Thr790Met) and p.(Cys797Ser) [12] and multiple mutations of *ALK* such as p.(Phe1174Leu), p.(Leu1152Arg) and p.(Cys1156Tyr) in lung cancers [13] and acquired resistance *EGFR* p.(Ser492Arg) in colorectal cancer [14]. Besides sequencing to stratify patients for targeted therapies, sequence data can also be used to refine histological diagnosis. Examples include differentiating between local tumour recurrence and secondary tumours in head and neck squamous cell carcinoma by sequence analysis of early tumour driver genes such as *TP53* [15] or the identification of clinically relevant groups of gliomas for diagnosis, prognosis and predictive testing by determining the presence or absence of *TERT* promoter mutations, *IDH1/IDH2* mutations and 1p/19q co-deletion [16].

Although employing whole genome sequencing for diagnosis may seem the ideal, it requires large resources leading to extended turnaround times and is not yet optimised for the use of fragmented and modified DNA and RNA isolated from FFPE material. The lower read depth also makes it very difficult to attain the sensitivity needed for routine sequencing of solid tumours in which genomic alterations might be present only in a small proportion of cells in the tumour tissue (neoplastic cell content) or neoplastic cells (tumour heterogeneity). Moreover, the genome-wide approach identifies large numbers of variants of unclear significance which, for adequate interpretation, requires concomitant analysis of germline DNA. This then increases the chance of finding incidental germline mutations, necessitating pre-test information sharing and informed consent procedures. More information on managing incidental germline findings can be found in references [17, 18]. Whole exome sequencing drastically reduces the sequencing and bioinformatics workload, but shares many of the drawbacks of whole genome sequencing and is not yet in routine use for molecular characterisation of solid tumours.

Because of the poor quality (due to fragmentation) and small amount of DNA extracted from FFPE, amplification-based enrichment methods using small amplicons are currently more successful than probe-based capture methods in this context.

The incorporation of a random barcode in PCR products enables the amount of sequenced template molecules to be determined and, with accompanying bioinformatic strategies, can reduce the background noise inherent in each NGS platform, allowing for a higher degree of confidence in individual result calls and for an increase in overall sensitivity.

The sequencing can be performed on different platforms which vary in capacity, turnaround time, level of automation and sequence technology. A summary comparison of methods can be found in Table 1. Most sequence platforms are based on sequencing by synthesis i.e. the sequential incorporation of fluorescent nucleotides of PCR products anchored on a glass slide (Illumina) or the release of protons after the incorporation of unlabeled nucleotides (Thermo Fisher). Each has its own artefacts. However, as the techniques are optimised over time, the number of artefacts will be reduced.

**Table 1:** A comparison table of NGS platforms and chemistries and discussion of specific applications

	Commercial panels	Custom panels	SNV	CNV		Indels	Fusions	DNA input	FFPE suitability	Comments
Whole genome	Y		Y	Y		Y	Y	1μg	Good-quality FFPE samples required to obtain DNA suitable for WGS	Hypothesis-free scanning of whole genome. Less sensitive for subclonal variation studies
Exome	y			y		Small			Good-quality FFPE samples required to obtain DNA suitable for exome sequencing	
Illumina Truseq cancer panels	Y	Y		Y	N	Small	N	10 ng +, more for FFPE	Yes, but requires good-quality material	Requires higher quality DNA than other equivalent technologies. Deep sequencing in a cost-effective manner. Identification of rare mutations andsubclonal variation detection in heterogenous tumour samples.
Illumina RNASeq				Y	Y	Small	Y	20 ng +	Yes	
Ampliseq cancer panels—DNA	Y	Y		Y		Small	N	10 ng +, more for FFPE	Yes	Larger panels require multiple oligos pool, homopolymer errors
Ampliseq cancer panels—RNA	Y	Y		Y	N	Y	Y	10 ng+	Yes	

## Implementation of NGS in a diagnostic laboratory

Test development and test validation, prior to the implementation and use of any NGS-based diagnostic test, is important. The implementation phase should develop an end-to-end process for testing and documenting the process being validated. Consideration should be given to the definition of suitable samples, appropriate extraction methods, quality control of test samples, fitness of the assay to cover clinically relevant variants to a sufficient depth for variant calling and the optimisation of the bioinformatics pipeline to detect relevant mutations at a suitable sensitivity. If there is insufficient coverage of a clinically important variant, re-design of a panel or commitment to test by alternative methods may be necessary. For molecular laboratories without developmental capacities, the use of commercially available pre-tested gene panels may be the best option. Nevertheless, testing in the local laboratory environment prior to the implementation and use of new gene panels using NGS should be standard for every laboratory.

The implementation phase can utilise samples with a range of known mutations and commercially available controls, but should be of the same type as those to be used for diagnostic testing. A range of mutation types must be tested, such as single-nucleotide variants, small indels and copy number variants (if tested for) to provide assurance that they will be detected if present. A range of allelic frequencies must be included so limits of detection for mutation types are established. Testing samples from the full range of clinically relevant samples should establish that the DNA or RNA can be extracted to suitable concentrations and quality for the required test and that the samples do not contain substances that may interfere with the assay.

## Validation and verification

### Validation

Validation involves defining the performance specifications of a test that are required to be met and generating evidence against these that demonstrates that appropriate test performance has been met: test accuracy, limitations and uncertainty of results. These criteria will depend on the intended use of the test as this defines how the test should perform. Therefore, the user of the test and their needs should be clearly identified.

Validation should test, end to end, all permissible sample types and should confirm the reliability of the assay to detect known mutations [19]. The validation procedure must also be fully documented and reviewed. The test performance characteristics selected e.g. analytical sensitivity, specificity, repeatability, reproducibility and robustness, should be measured for each sample type and for the range of mutations covered by the assay e.g. single-nucleotide variants (SNV), copy number variants (CNV), insertions and deletions. The number of variants tested should reflect the size of the panel. The region or genes to be covered by testing should be clearly indicated.

Validation studies should be performed blind with respect to the sample variants. Reference materials can be used; these may comprise commercially available samples that contain a range of mutations at varying allele frequencies or they may be samples with pre-defined mutations or mutations that will be confirmed later, by another method. To test the assay limit of detection (LOD), it may be necessary to dilute one sample into another in order to produce a range of mutations with varying allele frequencies. Validation samples should be representative of the material to be tested i.e. tests on FFPE-derived DNA should use validation samples that are also derived from FFPE tissue and extracted by the same method that will be used for diagnostic testing.

For somatic sequencing, LOD, expressed as a minimum variant allele frequency (VAF), should be defined along with the minimum coverage of sequencing reads to ensure that genomic alterations are reliably detectable. If several sample types are to be tested, separate LOD levels should be defined for each sample type.

In molecular pathology of solid tumours, 300 to 500 sequence reads per target are usually sufficient to cover almost all diagnostic situations if derived from sufficient template molecules. In a given sample, the exact number of reads required will depend on the neoplastic cell content of the sample as well as the VAF. For example, a sample with 40 % neoplastic cells where the test aims to detect mutations down to a VAF at 10 % will need much higher coverage than a 90 % neoplastic cell sample whose aim is to detect mutations at 30 % VAF, and the defined thresholds need to take this into account.

Statistical methods can be applied to predict if sufficient reads are available to call the genetic variants with confidence. Such statistics are only valid for hybridisation-based methods or if a molecular barcode is used. They are not applicable for the commonly used amplicon-based methods. A written validation protocol and defined acceptance criteria for samples should be developed for the entire workflow and adhered to for validation testing. Additionally, validation of any upgrades to the NGS analysis pipeline should be performed by direct comparison with the previous version.

The absolute number of samples required to ensure confidence and therefore validation of the test depends on the test being performed and the intended clinical use of the test results. This depends on factors such as the variants/panel under assessment, the region of the gene and how the variants will be used clinically [20]. It is important that the specific testing situation is taken into account. Not all possible allelic variants are available in the validation process. Therefore, a sufficient number of well-characterised samples should be used such that confidence is gained that within a region of interest the mutation is detected, thus the test should be challenged with at least one mutation variant with allelic frequency close to the LOD and with mutations that are notoriously difficult for the bioinformatic pipelines e.g. indels affecting multiple nucleotides A test is not validated when any control sample of known characterised mutation is not detected in which case the test should be modified and the process repeated. Possible sources of well-characterised samples for use in validation are listed in Supplementary Table 1 (ST1).

In order to validate a test and to perform ongoing monitoring of quality, performance characteristics of the NGS need to be measured and evaluated. Table 2 outlines the key test performance characteristics (TPC) for NGS used in molecular pathology.

**Table 2:** Test performance characteristics (TPC) of NGS relevant to molecular pathology

Test performance characteristic	TPC applied to NGS	Metrics and notes on assessment
Reportable range	The region of the genome in which a sequence of acceptable quality can be derived (may not be a contiguous region)	Reporting range must be confirmed during test validation
Reference range	The spectrum of normal variation of sequence within the population that the assay is designed to detect.	Test results outside this range may be clinically significant and require additional investigation.
Limit of detection (LOD)	The lowest allele frequency to which the assay can detect with an acceptable quality to enable confidence in a result i.e. the LOD is within the reporting range, (establishes the detection limit for sequence variants)	Minimum and maximum amount of DNA for 95 % test runs with adequate “no call” rate Allelic read percentage Sensitivity of the assay must be defined within the reporting range of allele frequencies and amount on input DNA for which the LOD was defined.
Repeatability	Concordance of variant detection between runs from the same sample under the same conditions e.g. prepare different libraries from the same samples run at the same time with the same operator and same instrument (within-run or intra-batch variability)	Analyse adequate number of runs depth of coverage Uniformity of coverage Transition/transversion ratio Pair-wise agreement
Reproducibility	Consistency of results from the same sample under different variations in conditions e.g. between different runs, different sample/library preparations, by different operators, or using different instruments (between-run or inter-batch variability).	Analyse adequate number of runs Depth of coverage Uniformity of coverage Transition/transversion ratio Pair-wise agreement
Accuracy (if reporting VAF)	The degree of agreement between the nucleic acid sequences derived from the NGS assay and a reference sequence (a measure of sequencing accuracy and error rates)	Adequate depth of coverage Uniformity of coverage Positive percent agreement Negative percent agreement Technical positive predictive value Rate of “no call” Allelic read fraction (number of independent reads assessed when calling a variant)
Precision (if reporting VAF)	The degree of agreement between replicate measurements of the same material across users and runs (a combination of reproducibility and repeatability)	Analyse adequate number of samples Depth of coverage Uniformity of coverage Transition/transversion ratio
Analytic sensitivity	The proportion of samples that test positive for a sequence variation and are correctly classified as positive (=TP / (TP + FN) (false-negative rate)	Depth of coverage Number of independent reads used to make a base call. This is dependent upon the amplifiability of the template DNA in the assay. Evaluation of base quality scores and signal-to-noise ratios
Analytic specificity	The proportion of samples that test negative for a sequence variation and are correctly identified as negative (=TN / (TN + FP) (false-positive rate) Some laboratories establish specificity by calculating the number of false positives per assay run.	Coverage (read depth and completeness) Number of independent reads assessed when making a base call Evaluation of base quality scores and signal-to-noise ratios. Potential for cross-reactivity and interfering substances Cross-contamination
Sequencing depth and allelic frequency cut-offs	The minimum sequencing coverage necessary for confident detection and variant calling (established for different variants)	

Adapted from [21], [22] and [49]

### Verification

Verification is the process that ensures existing, pre-defined, performance specifications are met in the local testing environment and hence the test performs as expected. This is in contrast to validation where the test performance specifications are defined.

NGS machines are available as a technology platform that can exploit various gene mutations as biomarkers for diagnosis, prognosis and selection of treatment and are therefore somewhat different to closed-system regulatory-approved kits that are developed for a specific purpose. For the latter, verification is important as regulatory-approved kits come with pre-defined performance specifications. In the case of NGS as an open platform, verification might be slightly different but may still be applicable in the case of methodology transfer e.g. adopting a protocol from another laboratory or a publication or for ensuring that a previously validated test is still performing as expected. Criteria for the expected performance of the test and what may lead to result rejection are considered in the “Quality assessment” section.

## Sample handling

The quality and representative nature of the samples analysed by NGS is a key limiting factor, an important issue addressed in many publications, and the processes specific to NGS are discussed below [8–14, 20–26].

### Sample transportation, receipt, handling and storing

The correct handling of tissue samples is key to obtaining a reliable result and is especially important for NGS as the quality of the DNA/RNA sample is critical (see “Quality assessment” section). Neutral buffered formalin should yield sufficient quality nucleic acid. The length of time of tissue fixation should be calibrated to the size of the specimen. The time between specimen removal and fixation, known as cold ischemia, alters DNA, RNA and protein expression, hence the need for controlled fixation of tissue [27]. In cutting sections for molecular analysis, the risk of cross-contamination can be minimised by replacing the knife regularly, avoiding water baths and using disposable plastic ware. Decontamination procedures that compromise nucleic acid yield or amplifiability should be avoided [28]. Cytological material that has been successfully tested for routine genotyping is recommended for molecular analyses in cases where tissue material is lacking and can often provide a high yield of quality DNA [17–20, 29–32].

The importance of pre-PCR processing is illustrated by a recent external quality assessment (EQA) ring study which found that across 13 molecular pathology laboratories, the rate of failure of mutation detection in *BRAF* and *EGFR* FFPE samples was 11.9 %, of which 80 % were attributed to pre-PCR errors [33].

### Morphological assessment and choice of region of interest

The histopathological diagnosis is mostly from H&E-stained sections of FFPE tissue. Morphological diagnosis and assessment of the fraction of neoplastic cells in tissue and cytological material are vital to the correct interpretation of NGS results. Once the diagnosis and representative nature of the material is established, it is recommended that the pathologist mark an area for extraction which contains an enrichment of neoplastic cells suitable to the level of detection i.e. appropriate allelic frequencies of genetic changes needed for the intended analysis. The selected fraction should be documented. Microdissection is not always required e.g. when the specimen contains a very high neoplastic cell content. To optimise DNA quality, the pathologist should try to avoid necrotic regions.

### Nucleic acid extraction, quantification and storage

In clinical practice, quality-controlled reagents for nucleic acid extraction are required. DNA extraction can be performed manually, in some instances using precipitation, but usually with the help of silica-based, commercial spin columns. All extraction protocols require strict adherence to ensure required yield and to avoid contamination or misidentification. There are also several validated automated systems, many which use magnetic bead-based extraction. These systems minimise hands-on time and lower the risk of misidentification. Commercial products that allow enzymatic removal of cytosine deaminations during the extraction step have recently been developed; these make DNA especially suitable for NGS applications (Qiagen). In the case of decalcified specimens, often a limited yield of good-quality DNA is obtained, therefore validation of the quality and quantity of DNA is a particularly important step for these samples. Furthermore, a recent data suggests that using EDTA rather than hydrogen chloride (HCL) to decalcify bone samples prior to embedding may improve the yield of usable nucleic acid for downstream NGS profiling [34]. Whatever system is selected, it is important to recognise that DNA and RNA yield and quality can vary between sample types.

Nucleic acids can be quantified spectrophotometrically (e.g. NanoDrop), fluorometrically (e.g. Qubit) or by quantitative PCR (qPCR). Combined quantification and fragment analysis can be performed by capillary electrophoresis of fluorescently labelled nucleic acids (e.g. BioAnalyzer). Assessments using PCR have the advantage of better prediction of the general amplifiability of the material, the key factor for downstream NGS analysis. Fluorometric assessments correlate fairly well with qPCR data and are generally recommended for use prior to NGS analyses along with qPCR, while spectrophotometric assays measure all nucleic acids and therefore overestimate double-stranded DNA concentration.

Nucleic acids are stored under highly controlled conditions in order to maintain sample identity and integrity. Sample accession logs or barcoded vials help avoid misidentification. Extracted DNA should be stored long term at −20 °C and RNA at −80 °C. Sequencing libraries and PCR products may be stored at either −20 °C or at −80 °C, but should be kept in separate freezers to avoid amplified material from contaminating non-amplified samples. The effects of length of storage of nucleic acids for NGS analyses has been insufficiently studied, but DNA or cDNA is more stable than RNA, and if stored appropriately, the integrity of the samples is more likely to remain intact.

### Sample misidentification

The verification of sample identity is essential in order to ensure data integrity and validity of conclusions and to assign results to the correct patient. The NGS workflow is a multistep process with inherent risks of sample mix-up, therefore procedures must minimise the risk of the misidentification, from the operating theatre to the reporting of the result. To this end, barcoding may be used and, if so, should be introduced into the pathway at the earliest opportunity. Panels can be designed to incorporate SNPs that allow molecular identification of patients and allow laboratories to monitor the incidence of sample switching by detecting chromosome X- and Y-specific sequences [35]. With different options available, ultimately it is important to perform a full end-to-end analysis of the risk of misidentification of the samples prior to introducing NGS and also at regular intervals during operation.

## Library preparation

Library preparation is the means by which genomic DNA is prepared for sequencing. The exact library preparation method will depend upon the sequencing platform and assay selected, the details of which are available from the manufacturer. Common to most platforms, the DNA must be fragmented and platform specific adaptors are added to the ends of the fragments. Targeted sequencing uses either an amplicon-based approach in which the DNA fragments are generated through short amplicons or through a hybridisation approach where a sequencing library is prepared for the whole genome, but only the regions of interest are kept for sequencing. Library preparation usually adds a molecular barcode to each DNA fragment, enabling identification of the sample following sequencing. The molecular barcoding also allows multiple samples to be pooled, reducing the costs involved and avoiding misidentification of samples.

To avoid contamination when preparing sequencing libraries, all steps prior to amplification should be carried out within a designated pre-PCR area containing dedicated equipment and reagents. Equipment should be fit for purpose and regularly maintained and calibrated. Reagents should be stored appropriately and used within the manufacturer’s expiry date. Extra care is required during tube transfers, and procedural training should underscore the avoidance of cross-contamination or accidental sample mix-up.

Following library preparation, the sequencing libraries should undergo quality control. This may include quantification, fragment size analysis and qPCR, using adapter sequences for priming to ensure that the sequencing library has been correctly formed and ensuring the appropriate positive and negative controls.

## Quality assessment

### General considerations

As NGS assays involve various combinations of instruments, reagents and bioinformatic pipelines, standardisation is challenging. Therefore, NGS-based assays require thorough assessment of potential pitfalls in different phases of the test cycle. As with any laboratory test, they are prone to sample contamination, sample mix-ups and tumour-normal switches. Once the validation work has been completed, continuous performance monitoring ensures that the test remains fit for purpose and detects quality issues before reporting a result. External Quality Assessment (EQA) schemes should be used wherever possible, and inter-laboratory comparisons may offer a suitable alternative where EQA schemes are not yet available.

### Next-generation sequencing quality scores

NGS quality scores will differ between sequencing platforms. Most sequencing platforms use DNA sequencing quality scores based on the Phred quality scoring system [36, 37]. Phred quality scores (Q scores) are logarithmically related to the probability of base calling errors. Historically, Phred scores have been used to determine the quality of Sanger sequencing; after initial base calling, Phred calculates parameters related to peak shape and resolution in relation to the base call, and a quality score is then assigned. Quality (Q) scores can range from 4 to 60, with higher values corresponding to higher quality (Table 3).

**Table 3:** Phred quality score table

Phred quality score	Probability of incorrect base call	Base call accuracy
10	1 in 10	90 %
20	1 in 100	99 %
30	1 in 1000	99.9 %
40	1 in 10,000	99.99 %
50	1 in 100,000	99.999 %
60	1 in 1,000,000	99.9999 %

NGS quality metrics vary from Sanger sequencing methods as there are no electropherogram peak heights to refer to. Parameters relevant to the particular sequencing platform are analysed for accuracy; using quality score tables, a quality score is calculated for NGS data which possesses an equivalent meaning to the Phred scores produced when using Sanger sequencing platforms.

Phred quality scoring is widely accepted as a method of assessing the quality of sequencing data including NGS data. Additionally, the Phred scoring system can be used to compare sequencing methods and platforms. Phred quality score analysis is easily integrated into automated data processing or the bioinformatics pipeline and will be utilised by the software analytics to assess the quality of a sequencing run alongside other relevant quality metrics specific to the platform.

### Quality assurance for NGS laboratory testing and bioinformatics

Recent guidance on quality control and recommendations for the use of NGS in different applications has been released [8–14, 20–26]. This guidance has been summarised in Table 4, acting as a checklist for the items and quality control (QC) metrics that require reviewing in order to provide confidence that the results are of sufficient quality to be released for clinical use.

**Table 4:** Recommended items to check prior to releasing NGS results for diagnostic use and QC metrics

Checklist item	QC metric(s)	Conditions when recommended to repeat or recheck before release	Consequences	Troubleshooting suggestions
Tissue sample acceptance criteria	Establish and document criteria for accepting or rejecting specimens. Minimum neoplastic cell content met? Sufficient amount of material provided? Suitable material for assay (FF or FFPE)? Tissue fixed appropriately? Transport conditions met?	If tissue fails to meet minimum neoplastic cell content or the test has not been validated for the type of tissue provided	Can lead to false-negative test results (failure to detect mutations present) and therefore sub-optimal treatment May result in re-testing that depletes available tissue sample for other tests	Requesting additional tissue samples is invasive for the patient and may not be possible. However, this should be highlighted in the report and a further sample requested (if clinically relevant).
Nucleic acid preparation	DNA quality (concentration, quality and volume) i.e. sufficient amount of amplifiable DNA? DNA quantity (double-stranded DNA content)	If the DNA falls below the minimum quantity or quality range that the test has been validated for	DNA that is not clean (e.g. trace EDTA or other inhibitors) can inhibit reactions. Can result in poor quality sequencing data, lower number of reads with adequate quality and variable recovery of sequence reads with high GC-content May lead to false-negative results and result in sub-optimal treatment. Tissue material may be depleted by multiple preparations.	Consider using a different extraction method: it is often helpful to have more than one extraction method validated for clinical use within the laboratory. Failed samples should be reported as such and further material requested, if clinically appropriate.
Sample identification	Sample is clearly identifiable	If there is not complete traceability of the sample identity	Misidentification of samples could lead to administering the incorrect treatment to a patient (exposing a patient to unneeded treatment or accidentally withholding a treatment that is required)	If it is not possible to trace a sample and there is concern that a sample may not come from the patient, then a further sample may be needed. Use of SNP or other identification methods to link the patient to the sample is now available in cases where this is a major issue and clinically indicated.
Library preparation	Minimum library concentration satisfied? Library preparation QC? Negative and positive controls in library preparation successful?	Check library QC for each sample and repeat or discard results if not acceptable.	May lead to poor or inaccurate coverage, especially in GC-rich regions. Library may have poor representation (lack the complexity) or bias not in the original sample. Can result in accuracies and failure to detect mutations present, which may result in sub-optimal treatment. There is also the possibility of false-positive results due to low allele frequency.	Consider new library preparation and/or use of an alternative method, such as PCR to verify uncertain results that are of clinical significance.
Sequencing	Minimum sequencing depth satisfied? Minimum sequencing quality metrics satisfied? Verify the quality of base calling and sequencing accuracy (e.g. minimum base quality Q score). Adequate average read length Coverage reviewed per sample, gene, exon and mutation to be reported	If fails to meet sequencing acceptance criteria then either limited reporting on genes reaching criteria or samples should be repeated	Less depth or coverage means more uncertainty in the integrity of the data. Limited reports may be acceptable if all actionable mutations are covered at sufficient depth, but consider repeating assay.	Repeat sequencing with existing library, or if indicated, start afresh with new sample. Verification of uncertain results with another method (e.g.) targeted PCR may be helpful if a clinically relevant finding is involved.
Variant detection and reporting	Variant present at allelic frequency and at adequate sequencing depth and quality score? % Reads mapped to reference genome, target region and % target covered Confirm presence of variant in forward and reverse strands. Check for non-specific mapping. Review mutation calls in the background of sequencing artefacts	Check presence and interpretation of variants found using validated software tools.	Variants of clinical significance may be missed or called incorrectly.	The best option here is probably to verify the finding with another method, if available, or to repeat sequencing.
Exceptions to standard protocols	Review impact of any documented exceptions to standard pperating procedure	Quality management issue: highlight non-compliances and eradicate from practice where possible.	Variants of clinical significance may be missed or called incorrectly.	Repeat the test using the existing library or stored DNA depending upon where in the process the error occurred.
Equipment and reagents	Reagents all within expiry and stored appropriately? Equipment appropriately maintained and calibrated?	Quality management issue: highlight non-compliances and eradicate from practice where possible. SOPs for reagent storage and regular servicing of equipment.	Poor reagent storage can lead to poor enzyme activity and poor ligation. Contamination can modify the end structure of the DNA and inhibit ligation. Variants of clinical significance may be missed or called incorrectly.	Order new reagents, and ensure expiry of consumables is checked before each run. Checklists can help ensure that this issue is avoided. For affected samples, repeat the test using the existing library or stored DNA depending upon where in the process the error occurred.
Bioinformatics	Correct pipeline and version run? Appropriate reference sequence used? Successful calling of any controls? Potential cross-contamination? Any tumour-normal pairs are appropriately matched?	Check presence and interpretation of variants found using validated software tools.	Newer analysis software may be better optimised. Using outdated or incorrect software can lead to variants of clinical significance which may be missed or called incorrectly.	Ensure software is updated regularly and that servicing agreements are in place for sequencers and associated equipment.
Results sign off	Result signed off by competent individual?	Ensure that all those signing off reports have evidence of adequacy of training and competence	Variants of clinical significance may be interpreted or presented incorrectly, leading to incorrect interpretation and treatment.	Ensure competence of individuals analysing and reporting NGS results: training records should be maintained regularly.

All assays should be validated for the sample type and the conditions in which the assay is run. Working outside validated conditions, e.g. different samples, reagents and working environment, increases the risk of false positives and negatives. For clinical runs, wherever possible, manufactured negative and positive controls which contain a large amount of variant types should be run; however, for practical purposes, this is not always possible but it is recommended to run a positive control sample. The performance of the control can be monitored over the test, determining that the same variants are detected each time.

When parameters are near acceptable limits, professional judgement should be used to determine if the result should be reported.

The bioinformatics methods should be validated to detect the range of mutations for which the assay has been designed, in a range of sequencing contexts and within the accepted range of conditions for the assay.

## Data analysis of sequence reads

The combination of bioinformatics tools used for processing, aligning and detecting variants in NGS data is commonly referred to as the data analysis or the bioinformatics pipeline. Currently, no single algorithm can analyse all types of sequence variants; therefore, different software tools often are applied to NGS data in order to answer the clinical question. The difficulty in designing uniform and transferable pipelines ultimately requires each laboratory to validate the bioinformatics pipeline in accordance with the types of variants to be reported. Commercially available software packages especially designed for the purpose of analysing amplicon-based NGS data have a significant advantage over individual and homemade bioinformatics solutions.

### Read processing/read quality

The initial step in the analysis pipeline is usually performed by the sequencer, converting the sequence into digital information in order to be further processed. This information is later used for the variant calling process.

### Alignment

This step involves the alignment of sequencing reads to the genome of reference. In the study of genetic alterations in solid tumours, the human genome reference is the standard. Alignment reference can utilise either the entire genome or the genomic regions of interest (ROI). If the whole genome is used, the computational time will be longer than the use of specific ROI, while on the other hand, the use of ROI forces the algorithms to align the reads to those regions which may result in lower-quality calls.

NGS generally produces short reads of approximately 80–200 bases. As the reads are equally short, alignment problems are introduced, for there may be several equally likely places in the reference sequence from which they could have been read. This is especially true for large deletions or insertions, repetitive regions and pseudo or homologous genes, the latter being especially problematic if reads are mapped to an ROI instead of the whole genome.

Mapping algorithms also need to account for different types of technical error. As many NGS methods involve one or multiple PCR steps, PCR errors will be shown as mismatches in the alignment. This particularly applies to errors in early PCR rounds which will show up in multiple reads, falsely suggesting genomic variation in the sample. Related to this are PCR duplicate errors which occur multiple times in the same read, changing coverage calculations in the alignment. Sequencing errors result from the sequencing machine making an erroneous call, either for physical reasons, e.g. dust on the flow cell, or due to the particular properties of the sequenced DNA e.g. homopolymers. These sequencing errors are often random so they can be filtered out as single reads during variant calling.

Still, although alignment algorithms must be fine-tuned, they need to allow a certain level of mismatch; otherwise, no variation would ever be observed. This is especially true in the context of somatic alterations where a variant might be present in only a subgroup of neoplastic cells, introducing the possibility that a variant may be present at differing allelic frequencies from very low to very high, in stark contrast to germline alterations that are present with 50 % heterozygous or 100 % homozygous allelic fractions.

### Variant calling

The quality of a variant call is strongly related with the quality of the alignment e.g. the more relaxed the alignment algorithm is, the more variants can be potentially called. The key challenge of variant caller algorithms is to help distinguish sequencing errors and call “real” variation. Therefore, there is a direct effect of read depth; the more times a variant is sequenced, the more reliable the call. Here, the difficulties already described for the correct identification of variants with low allelic fractions also play an important role. The minimum depth of coverage depends on the required sensitivity of the assay, sequencing method and the type of mutations to be detected. The algorithm parameters should be varied during assay development in order to derive optimal settings for each variant the test is designed to detect. Laboratories should consider the implementation of modular analysis pipelines in which different algorithms or settings are used to analyse the same data set and to call the different types of variants, i.e. SNV, insertions, deletions and copy number alterations. In the absence of a ‘gold standard’, bioinformatics pipelines should be extensively validated [38–40].

### INsertion/DELetion (indels)

Indels (INsertion/DELetion) are the second most common type of genomic variation, and certain indels have proven to have clinical relevance such as in *EGFR, KIT* and *ERBB2*. The reliable identification of indels by software packages post-sequencing has proven challenging due to insufficient and inaccurate mapping to the reference sequence. This is compounded by the fact that indels occur at a lower frequency than other variants which makes distinguishing alignment artefacts and sequencing errors (especially in homopolymer regions) more problematic. As a result, the sensitivity and specificity for this type of variant is often reduced and high false-positive rates are noted.

Laboratories must identify the clinically important indels which are targeted within the scope of their particular assay and validate these accordingly. The validation process should enable the laboratory to establish both a sensitivity and positive predictive value for indels. These values will depend on the type of sequencing technology and the bioinformatics/alignment algorithm used. Due to the high rate of false positives, it is recommended that all identified indels are confirmed by manual visualisation tool such as the Integrative Genomics Viewer (IGV).

### Variant annotation and filtering

Annotation of sequence variants determines if a sequence variant is either false or true and if the functional interpretation of that variant is related to the gene (protein) function. False variants are artefacts, variants that are absent from the in vivo tissue and that are introduced during the pre-analytical process or during storage of the specimen. There are three major causes for false sequence variants: DNA deamination artefacts, amplification errors and sequencing errors. A major cause for false variants in FFPE tissue is due to deamination of cytosine bases resulting in C:G>T:A substitutions during amplification. Hydrolytic deamination occurs naturally, so older specimens will be more prone to these types of errors, but formalin fixation contributes to this process [41]. Since these variants are usually present at very low frequency, such artefacts are unlikely to interfere with data analysis if enough unique DNA molecules are available during NGS library enrichment. However, if the number of unique DNA molecules is limited or if the aim is to detect low-level variants, these artefacts will interfere with data analysis. Since deaminations are introduced during processing of the sample ex vivo, these variants are not copied into the DNA, meaning that the variant is present on only one sense or antisense DNA strand.

To facilitate detection of fixation artefacts, several techniques independently enrich and/or sequence both the sense and antisense DNA strands e.g. molecular inversion probes and HaloPlex and Duplex sequencing. Treatment with uracil-DNA glycosylase prior to PCR amplification markedly reduces these artefacts without effecting true mutational sequence changes [41, 42].

Another source of false variants is introduced during the amplification steps of library enrichment. If sufficient unique DNA molecules are present, the effect of polymerase errors will be limited, posing a problem for the detection of low-level variants. One solution is the sequencing of single or unique molecules. Library enrichment techniques are available that allow barcoding of individual DNA molecules. Since polymerase errors occur during amplification, it is unlikely that individual DNA molecules will independently acquire the same polymerase error during amplification. The detection of the same variant in multiple unique molecules therefore allows for the discrimination between ultra-low level variants and polymerase artefacts.

Also, false variants can be introduced during the sequencing process itself. The rate of such sequencing errors is highly dependent on the sequencing platform and the chemistry used. The representation and ability to call single base variants and indels are similarly accurate for data generated on the PGM and Illumina platform, provided there is sufficient coverage. Sequencing of homopolymers using the PGM platform showed a higher indel calling error rate. However, base/variant calling in the Torrent Browser and using the newer versions of Torrent Suite Variant Caller have shown significant improved accuracy especially for indels in homopolymers up to eight bases [43, 44]. It is recommended to use proper computational indel calling analysis of variants in clinical relevant homopolymers to maximise both the sensitivity and specificity at the single base level. In addition, all identified indels at homopolymers should be confirmed by a manual visualisation tool such as IGV and directly compared to sequence data of the same regions in other samples analysed in the same run.

### Variant interpretation

The next step is the functional annotation and subsequent biological interpretation of the true variants. Depending on the type of variation (e.g. non-sense versus missense) or on the type of gene (e.g. hotspot position in an oncogene versus tumour suppressor gene), this can be either a simple or very laborious process. Since interpretation of genetic data becomes more complex, the ACMG strongly recommends that interpretation is performed in the laboratory by suitably trained staff such as clinical molecular geneticists, clinical scientists in molecular pathology or molecular pathologists [45]. Furthermore, a multiple disciplinary team approach from technical, scientific and clinical members will enable appropriate clinical interpretation of the results in the context of current drug and clinical trials.

Multiple sources of information are required to describe variants of uncertain significance (VUS). When sequencing DNA from tumour tissue, an effective way to discriminate somatic variants from germline variants is to sequence, in parallel, reference DNA from non-neoplastic tissue from the same person. Otherwise, the first step is to retrieve the population frequency of the VUS from the dbSNP database to discriminate common variant polymorphisms from other unknown variants (UV). Although there is no clear cut-off, UVs with a population frequency >1% are usually considered to be polymorphisms. Other databases e.g. Catalogue of Somatic Mutations in Cancer (COSMIC) or ClinVar (which aggregates information about genomic variation and its relationship to human health) can provide information regarding frequency and biological function of variants in disease. Further classification of VUS can be accomplished using algorithms that consider nucleotide conservation throughout evolution e.g. phastCons and phyloP and physicochemical difference between wild-type and mutated amino acids i.e. Grantham distance, or predict the possible impact of mutations on protein structure and function e.g. PolyPhen, SIFT, and Align GVGD. Examples of software solutions, databases and tools that facilitate variant classification and interpretation are presented in Supplementary Table 2 (ST2).

Based on the available data, e.g. variant frequency, predictions, knowledge bases and functional studies, a variant can be described using standard terminology e.g. benign, likely benign, of uncertain significance, likely pathogenic or pathogenic [46] or activating, neutral/VUS and inactivating [47].

Overall, the advantage and limitations of existing tools must be objectively evaluated by clinical or pathological laboratories according to their specific sequencing needs. For NGS data analysis, the acceptable thresholds for data quality and depth of coverage should be determined during the assay development and validation process. Quality thresholds should include metrics such as base calling quality, coverage, allelic read percentages, strand bias and alignment quality. Importantly, the thresholds validated for the detection of germline alterations cannot be transposed for the identification of somatic alterations in tumour samples [48].

## Reporting of results

### Content of test report

The reporting of NGS results should follow the general principles of clinical reporting and sit in line with international diagnostic standards such as ISO 15189 (2) and professional guidelines [8–14, 20–26] (see Table 5). It is essential that clinically actionable results are reported in a clear and consistent manner and the use of supplemental documentation [17] ensures the reader of the report is in receipt of all the information required to appropriately interpret the results. Clear reporting is also critical since laboratory reports may be read by both experts and non-experts. However, there are specific issues related to the reporting of somatic genomic variants, and these will be considered below.

**Table 5:** Information to be included on the NGS patient report

• Patient identifiers	
• Sample type e.g. FFPE, fresh frozen	
• Tissue/tumour type e.g. lung, colorectal, melanoma	
• Tissue sample identification e.g. unique block number	
• Restatement of the clinical question	
• Percentage of neoplastic content of sample used for NGS	
• Extent of testing performed i.e. the genes and more specifically which regions tested e.g. exons, introns or hot spots analysed	
• NGS method used e.g. platform, type of panel (amplicon, hybridisation), exome, WGS	
• Sensitivity of the method i.e. percentage variant alleles detectable in a background of wild-type DNA	
• Reference sequences for genes tested^a^	
• Results (using HGVS mutation nomenclature)^a^	
• How/where additional information about the analysis can be obtained^b^	
• Interpretation and conclusion (see “Interpretation” and “Other reporting considerations” sections)	

^a^It is essential to use Human Genome Variation Society (HGVS; http://www.hgvs.org/mutnomen/) mutation nomenclature and to include the appropriate reference sequence including version number used for gene or transcript number if locus-specific genome sequences (LRGs) are used. For reference purposed, the HUGO Gene Nomenclature Committee (HGNC) approved that gene symbols should be used at least once. In addition, it is strongly recommended to include genomic coordinates in order to ensure uniform bioinformatics analysis and consistent documentation of identified variants. If genomic coordinates are used, then the appropriate genome build must be stated. Exon annotation of the identified variants is not required, since version updates of the reference sequences occur frequently. However, the use of LRGs can help to avoid the changes in exon numbering

^b^In most cases, requesting clinicians will find the information on the standard patient report sufficient for their needs, but in some instances, supplementary details may be necessary. These could include technical characteristics of the test methodology, bioinformatics pipelines, validation reports and methods for annotation and classification of variants. How these supplementary details can be obtained should be stated on the patient report. Supplementary details may be in the form of a report, for example, “supplementary technical report available on request” can be stated on the patient report. Alternatively, the clinician could be directed to a controlled document such as a “laboratory handbook” or to a link for online information. In any case, care must be taken with document version control and online links should divert the user to data pertinent to the way the specific sample was tested and analysed, even if the methodology has been superseded for current samples

The NGS patient report should ideally be one page in length (if not possible, then no more than two pages) and contain the information outlined in Table 5. It is important that the most pertinent information, i.e. results and conclusions, are positioned prominently on the report and clearly visible to requesting clinicians. Sufficient technical information should be provided (Table 5), but the inclusion of in-depth technical information is not recommended. However, in some cases, service users may require more detailed methodological information; therefore, the report should contain clear information on how to access these details.

### Interpretation

The purpose of clinical diagnostic testing of solid tumours is to identify the presence or absence of variants, in order to aid diagnosis, predict prognosis or guide optimal therapy. Therefore, variants should be classified and reported according to their potential for clinical action and the robustness of the result. The robustness of a given variant for clinical utility falls into three domains:

1. Clinical—variants that have a current approved/licenced therapeutic indication or are used clinically for diagnosis, prognosis or therapeutic monitoring
2. Clinical trials—variants that are hypothesised to predict response to a novel compound and entry to a clinical trial may be possible.
3. Research—mutations not currently used to inform clinical management but have a biological effect implicated in tumour oncogenesis which may have future clinical utility.

There should be an agreement between the laboratory and its service users over which classification/domains of mutations should be included on patients’ reports. For example, domain 1 mutations should always be included; however, it would not be appropriate to list domain 3 mutations which have no current clinical utility on a routine diagnostic report. Alternatively, it may be useful to include domain 3 variants on a report being forwarded to a clinical research centre.

The mainstay of assessing the ability to action a variant should be via the usual literature searches. Due to the fast pace of cancer research and therapy development, constant vigilance and regular update of searches is required to ensure the advice given on reports is up to date. There is an increasing number of online resources such as mycancergenome.org which contain lists of tumour types, genes and variants and include assessments of clinical impact. These resources can be very useful, but before such a source is used, it is important to ensure that it is properly curated, referenced and regularly updated. In this regard, recent guidance from the FDA has suggested considerations for determining whether a genetic variant database is a valid source of scientific evidence for supporting the clinical validity of genotype-phenotype relationships that may assist laboratories in selecting appropriate data sources [49]. The guidance suggests users seek databases that implement decision matrices with published details supporting each variant’s interpretation as well as having documented standard operating procedures for outlining the process for curation of evidence and that demonstrate that multiple sources for evidence are used.

When assigning variants to domains, it is essential that they are interpreted in the context of the tumour/tissue type, as the resulting clinical action will vary e.g. mutations classified as domain 1 in a particular tumour type could fall into domain 2 or 3 in another tumour type. In other words, the classification of variants will be specific to the tissue and tumour type. When reporting domain 1 variants, a clear indication of their clinical relevance and any appropriate actions should be included. For domain 2 variants, it may be useful to alert the requesting clinician to the possibility that clinical trials may be available. Furthermore, as new evidence and therapeutics emerge, the classification of particular variants will change.

### Other reporting considerations

It is essential that NGS results are interpreted in conjunction with other pathology results from the sample, such as morphological assessment and immunohistochemistry. It is therefore recommended that the pathology report includes a subsequent integrated/supplementary summary which includes *all* the relevant test results and an overriding conclusion.

In cases where the neoplastic cell content of the sample is close to or below the analytical sensitivity of the method used, a remark alerting the clinician to the possibility of a false-negative result should be included.

Variant allele frequency should only be included on the patient report if the clinical significance of differing levels of allele frequency is known.

Laboratories should have a clearly defined protocol for addressing any unsolicited and secondary findings that may arise during testing *prior* to launching the NGS test.

In cases when the full laboratory quality standards have not been met but it is felt a limited interpretation can be made, then the limitations of the analysis should be made clear on the report.

## Electronic supplementary material

ESM 1 (DOCX 85 kb)

ESM 2 (DOCX 19 kb)
