# Supplementary material for: Integration of next-generation sequencing in clinical diagnostic molecular pathology laboratories for analysis of solid tumours; an expert opinion on behalf of IQN Path ASBL
Source: Virchows Arch. 2016 Sep 27;470(1):5–20. doi: 10.1007/s00428-016-2025-7 (PMC5243883; doi:10.1007/s00428-016-2025-7)
Supplement: Supplementary file 1 — (DOCX 85 kb) [file 428_2016_2025_MOESM1_ESM.docx]

Supplementary table 1 (ST1): Examples of sources useful for identifying samples to be used in validation of NGS methods.

| Sample | Source |  |
| --- | --- | --- |
| Vendors | Acrometrix | *https://www.thermofisher.com/uk/en/home/brands/product-brand/acrometrix.html* |
|  | Horizon Diagnostics | *https://www.horizondiscovery.com/reference-standards* |
| Known patient sample | Ask a colleague | *www.genome.gov/10001688/international-hapmap-project* |
| Cell line with known / engineered sequence variation(s) | ATCC | *http://www.lgcstandards-atcc.org/Products/Cells_and_Microorganisms/Cell_Lines.aspx?* |
| Patient samples | Contact biobanks | [*http://bbmri-eric.eu/bbmri-eric-directory-2.0*](http://bbmri-eric.eu/bbmri-eric-directory-2.0)  [*www.isber.org*](http://www.isber.org)  [*www.coriell.org*](http://www.coriell.org/) *(Genome in a Bottle sample)*  www.cap.org *(College of American Pathologists)* |
