# Supplementary material for: Integration of next-generation sequencing in clinical diagnostic molecular pathology laboratories for analysis of solid tumours; an expert opinion on behalf of IQN Path ASBL
Source: Virchows Arch. 2016 Sep 27;470(1):5–20. doi: 10.1007/s00428-016-2025-7 (PMC5243883; doi:10.1007/s00428-016-2025-7)
Supplement: Supplementary file 2 — (DOCX 19 kb) [file 428_2016_2025_MOESM2_ESM.docx]

| **Algorithm / Software tools** | **Company / reference** |
| --- | --- |
| VariantDB | Vandeweyer, et al. 2014 Genome Med. 6(10):74 |
| QCI | Qiagen |
| Cartagenia bench Lab | Cartagenia |
| Nextbio | Illumina |
| Alamut Visual | Interactive Biosoftware |
| Sophia Genetics | Sophia Genetics |
| Opal Clinical Genomics | Omicia |
| **Websites / databases** |  |
| MyCancerGenome | Yeh, et al. 2013 Clin. Cancer. Res. 19(7):1894-901 |
| DoCM (database of curated mutations) | http://docm.genome.wustl.edu |
| CIVIC | https://civic.genome.wustl.edu |
| COSMIC | Forbes, et al. 2015 Nucleic Acid Res. 43:D805-11 |
| ICGC | http://icgc.org/ |
| TCGA | http://cancergenome.nih.gov/ |
| HGMD | http://www.hgmd.cf.ac.uk, Qiagen |
| NGRL diagnostic mutation database | http://www.ngrl.org.uk/Manchester/projects/dmudb |
| 1000 genome project | <http://www.1000genomes.org/about>. |
| Café Variome | http://www.cafevariome.org/ |
| Locus-specific databases | Lists of LSDBs are available from <http://www.lovd.nl/2.0/index_list.php>, <http://www.hgmd.org/>, <http://www.gen2phen.org/data/lsdbs>, <http://www.hgvs.org/dblist/glsdb.html> |
| Missense Prediction Tool Catalogue | http://www.ngrl.org.uk/Manchester/page/missense-prediction-tool-catalogue |
| **Prediction Algorithms** |  |
| SIFT | Kumar, et al. 2009 Nat. Protoc. 4(7):1073-81  Ng, et al. 2006 Annu Rev Genom Hum Genet 7:61-80  Ng, et al. 2003 Nucleic Acids Res. 31(13): 3812-4  Ng, et al. 2002 Genome Res 12(3): 436-46  Ng, et al. 2001 Genome Res 11(5): 863-74 |
| Align-GVGD | Tavtigian, et al. 2006 J Med Genet 43(4):295-305 |
| Mutation Assessor | Reva, et al. 2011 Nucleic Acids Res 39(17):2118  Reva, et al. 2007 Genome Biol 8(11):R232 |
| PANTHER | Brunham, LR et al. 2005 Accurate prediction of the functional significance of nucleotide polymorphisms and mutations in the ABCA1 gene. PLoS Genetics 1 (6) e83. |
| MAPP | Stone EA et al 2005 Physicochemical constraint violation by missense substitution mediates impairment of protein function and disease severity. Genome Research 15 978-986 |
| Grantham Score | Grantham Science 1974 185(4154):862-4 |
| PolyPhen-2 | Adzhubei, et al. 2010 Nat Methods 7(4):248-9 |
| [LS-SNP/PDB](http://www.ngrl.org.uk/Manchester/page/ls-snppdb) | Ryan M., et al. 2009 LS-SNP/PDB: annotated non-synonymous SNPs mapped to protein data bank structures. Bioinformatics 25(11):1431-2. |
| [SNPeffect](http://www.ngrl.org.uk/Manchester/page/snpeffect) | De Baets G, et al. 2012 SNPeffect4.0: online prediction of molecular and structural effects of protein-coding variants.Nucleic Acids Research 40(1):D935-9. |
| Protein stability-based methods: MUpro, FoldX, PoPMuSiC, SDM | Listed here: http://www.ngrl.org.uk/Manchester/page/protein-stability-based-methods |
| SpliceSiteFinder | Zhang, et al. 1998 Hum Mol Genet 7(5): 919-32 |
| MatEntScan | Yeo, et al. 2004 J Comput Biol11(2-3): 377-94 |
| NNSplice | Reese, et al. 1997 J Comp Biol 4(3): 311-23 |
| GeneSplicer | Pertea, et al. 2001 Nucleic Acids Res 29(5): 1185-90 |
| Human Splicing Finder | Desmet, et al. 2009 Nucleic Acid Res 37(9): e67 |
| NetGene2 | S.M. Hebsgaard, P.G. Korning, N. Tolstrup, J. Engelbrecht, P. Rouze, S. Brunak: Splice site prediction in Arabidopsis thaliana DNA by combining local and global sequence information, Nucleic Acids Research, 1996, Vol. 24, No. 17, 3439-3452. Brunak, S., Engelbrecht, J., and Knudsen, S.: Prediction of Human mRNA Donor and Acceptor Sites from the DNA Sequence, Journal of Molecular Biology, 1991, 220, 49-65. |
| ESEFinder | Smith, P. J., Zhang, C., Wang, J. Chew, S. L., Zhang, M. Q. and Krainer, A. R. 2006.  An increased specificity score matrix for the prediction of SF2/ASF-specific exonic splicing enhancers. Hum. Mol. Genet. 15(16): 2490-2508.  Cartegni L., Wang J., Zhu Z., Zhang M. Q., Krainer A. R.; 2003.  ESEfinder: a web resource to identify exonic splicing enhancers. Nucleic Acid Research, 2003, 31(13): 3568-3571. |
| [PMut](http://www.ngrl.org.uk/Manchester/page/pmut) | Ferrer-Costa C., Orozco M., de la Cruz X. Sequence-based prediction of pathological mutations. Proteins (2004) 57 811-819 |
| [SNAP](http://www.ngrl.org.uk/Manchester/page/snap-screening-nonacceptable-polymorphisms) | Bromberg Y., Tachdav G., Rost B. SNAP predicts effect of mutations on protein function. Bioinformatics (2008) 24, 2397-2398. |
| [PhD-SNP](http://www.ngrl.org.uk/Manchester/page/phd-snp) | Capriotti E., Calabrese R., Casadio R. Predicting the insurgence of human genetic diseases associated to single point protein mutations with support vector machines and evolutionary information. Bioinformatics.(2006) 22 (22) 2729-2734. |
| [SNPs&GO](http://www.ngrl.org.uk/Manchester/page/snpsgo) | Calabrese R., Capriotti E., Fariselli P., Martelli P.L., Casadio R. Functional annotations improve the predictive score of human disease-related mutations in proteins. Hum Mut.(2009) 30 1237-1244. |
| [Parepro](http://www.ngrl.org.uk/Manchester/page/parepro-prediction-amino-acid-replacement-probabilty) | Tian J., Wu N., Guo X., Guo J. Zhang J., Fan Y. Predicting the phenotypic effects of non-synonymous single nucleotide polymorphisms based on support vector machines. BMC Bioinformatics (2007) 8 450-464. |
| [CanPredict](http://www.ngrl.org.uk/Manchester/page/canpredict) | Kaminker J.S., Zhang Y., Waugh A., Haverty P.M., Peters B., Sebisanovic D., Stinson J., Forrest W.F., Bazan F., Seshagiri S., Zhang Z. Distinguishing cancer-associated missense mutations from common polymorphisms. Cancer Research (2007) 67 (2) 465-473. |
| [nsSNPAnalyzer](http://www.ngrl.org.uk/Manchester/page/nssnpanalyzer) | Bao L., Cui Y. nsSNPAnalyzer: identifying disease-associated nonsynonymous single nucleotide polymorphisms. Nucleic Acids Research (2005) 33 W480-W482 |
| [MutPred](http://www.ngrl.org.uk/Manchester/page/mutpred) | Li B., Krishnan V.G., Mort M.E., Xin F., Kamati K.K, Cooper D.N., Mooney S.D., Radivojac P. Automated inference of molecular mechanisms of disease from amino acid substitutions. Bioinformatics (2009) 25 (21) 2744-2750. |
| [Hansa](http://www.ngrl.org.uk/Manchester/page/hansa) | Acharya V. and Nagarajaram H.A. Hansa: An automated method for discriminating disease and neutral human nsSNPs. Human Mutation (2012) 2:332-337 |
| [MutationTaster](http://www.ngrl.org.uk/Manchester/page/mutationtaster) | Schwarz J.M., Rödelsperger C., Schuelke M., Seelow D. MutationTaster evaluates disease-causing potential of sequence alterations. Nature Methods (2010) 7 (8) 575-576. |

Table ST2. Examples of (commercial) software solutions, databases and tools that facilitate variant classification and interpretation after variant calling. Solutions for mapping and variant calling are not included.
